# 

**Published:** 1883-09-20

**Authors:** 


### TABLE OF CONTENTS.

ORIGINAL AND SELECTED ARTICLES.

The Therapeutic Action of Minute and Frequently Repeated Doses of Medicines, by G. G. Roy, M.D., Professor of Materia Medica in Southern Medical College, Atlanta, Ga., 321 Oil of Gaultheria, by J. H. Logan, M.D., of Georgia, 325. An Unrecognized Cause of Gastro-Intestinal Irritation in a Child Discovered, by G. G. Roy, M. D.. Professor of Materia Medica in Southern Medical College, 326. Extracts from a Paper on the Prevention of Insanity, by Nathan Allen, M D., of Lowell, Mass. 328. The Therapeusis of Remittent Fever, by A. M. Pelton, M.D., of Texas, 331. Anæsthetics in Labor, by E. Herriott, M. D., Jacksonville, Ill., 334. Acute Laryngitis, by C. E. Nelson, M.D., N. Y., 335. Trance in a Hysterical Woman, 336.

ABSTRACTS AND GLEANINGS.

Salicylate of Bismuth in Typhoid Fever, 337. Reviving the Still-Born, 338. Malarial Hæmaturia, 339 Cephalhæmatoma in the New-born, 340. Treatment of Gout, 341. Examination of Urine; The Parasite of Marsh Fever, 342 Ante-Natal Murder; Cholera Morbus 343. Ipecac in Labor; How to Remove a Tight Ring, 344. Berberis Aquifolium in Syphilis, 345. A Case, 346. Rheumatism Aborted; What Means can be used to Lessen the Hours of Labor?; Note on Disinfectants, 347. Local Application of Vaseline in Scarlet Fever; Effects of the Internal Administration of Glycerine, 348. A New Remedy for Malarial Fever; Cannabis Indica in Menorrhagia; Ergot in the Treatment of Congestive Headache, 349. Did Syphilis Exist in America before the Discovery by Columbus?; Pruritus Ani; Vegetable Nature of Croup, 350.

SCIENTIFIC ITEMS.

Copperhead Venom; Inventor of the Telephone; Showers of Iron, 351. Household Ventilation; Variety of Colors, 352.

PRACTICAL NOTES AND FORMULÆ.

Hypophosphite of Lime in Cancer; For Torpid Liver, 353. Naphthol for Local Sweating; Pain Killers; For Excoriated Nipples, 354. Eczema of the Hands; Remedy for Migraine, or Hemicrania, 355.

EDITORIALS AND MISCELLANEOUS.

Notice; Editorial Notices; American Academy of Medicine, 356. Medical Ministry; Georgia Medical Association; Professional Secrets, 357. Rotheln; Book Notices, 358. Faith Cures; Pamphlets Received, 359. Special Notices, 360.

